# Safety of Antibiotics in Hospitalized Children in Romania: A Prospective Observational Study

**DOI:** 10.3390/ph15060713

**Published:** 2022-06-03

**Authors:** Noémi-Beátrix Bulik, Andreea Farcaș, Camelia Bucșa, Irina Iaru, Ovidiu Oniga

**Affiliations:** 1 Department of Pharmaceutical Chemistry, ‘Iuliu Hațieganu’ University of Medicine and Pharmacy, 400010 Cluj-Napoca, Romania; noemi.beatrix26@gmail.com (N.-B.B.); onigao65@yahoo.com (O.O.); 2 Pharmacovigilance Research Centre, ‘Iuliu Hațieganu’ University of Medicine and Pharmacy, 400349 Cluj-Napoca, Romania; cfarah@umfcluj.ro; 3 Department of Pharmacology, Physiology and Pathophysiology, ‘Iuliu Hațieganu’ University of Medicine and Pharmacy, 400349 Cluj-Napoca, Romania; aniris_aniris@yahoo.com

**Keywords:** adverse drug reaction, antibiotics, hospitalized children, causality, avoidability

## Abstract

Antibiotics are among the most prescribed drugs in pediatric inpatients and are frequently associated with adverse drug reactions (ADRs) in children. This study aimed to assess the frequency and type of ADRs related to the use of antibiotics in pediatric inpatients through a prospective observational study, conducted over 6 months, covering the winter and spring seasons when the incidence of infections peaks in Romania. ADRs were evaluated for causality, avoidability and severity. Among the 266 included children, 25 (9.4%) experienced 30 ADRs. ADR frequency tended to be higher in ≤2-year-olds (13 of 25, 52.0%) than in other age categories. Gastrointestinal and hematological ADRs were most frequently observed. Diarrhea was the most common ADR associated with antibiotics (8 of 30, 26.7%). Ceftriaxone (16 of 30, 53.3%), cefuroxime, ceftazidime and azithromycin (3 of 30, 10.0% each) were most commonly responsible for ADRs. After causality assessment, 2 (6.7%) ADRs were considered definite, 12 (40.0%) probable and 16 (53.3%) possible. One ADR was classified as definitely avoidable and one as possibly avoidable. Seven children required treatment for ADRs. Antibiotic treatment was discontinued in 4 children. Antibiotics frequently caused ADRs in ≤ 2-year-olds and were commonly associated with gastrointestinal symptoms. Close monitoring of antibiotic-associated ADRs remains important in the pediatric population.

## 1. Introduction

Antibiotics are widely prescribed to children in primary care and hospital settings [1,2]. Patterns of antibiotic prescription vary across different age groups [1] and in different countries according to national guidelines [3,4]. Antibiotics are responsible for almost a quarter of the adverse drug reactions (ADRs) occurring in hospitalized children; a higher incidence of ADRs was observed in hospitalized versus non-hospitalized children [5,6].

The low number of clinical trials conducted on children limits the amount of available data on drug safety for this category of patients [7]. In addition, not all ADRs experienced by adults can be extrapolated to children due to the state of developing organs and systems in the pediatric population, resulting in age-specific physiology [8,9]. Spontaneous reporting systems may be an important source of ADRs, but the reporting level of ADRs in children remains low in many countries, including Romania [8,10,11].

In 2019, Romania was among the top 3 countries with the highest rates of antibiotic consumption in Europe (25.8 defined daily doses per 1000 inhabitants per day) [12]. A study conducted in Romania showed that antibiotics were the most frequently prescribed drugs in pediatric inpatients; broad-spectrum antibiotics like cefuroxime, azithromycin, ceftriaxone and meropenem were frequently prescribed both on- and off-label, and often in a higher dose than recommended [13].

To the best of our knowledge, to date, there has been no study assessing ADRs caused by antibiotics in the pediatric population in Romania. Since the health care systems and prescribing patterns vary across countries, and therefore the findings of studies conducted in one country cannot be generalized for all, this study aimed to evaluate the frequency and characteristics of ADRs following antibiotic treatment in hospitalized children in Romania.

## 2. Results

Two hundred sixty-six children with 274 hospital admissions (4 children had 3 admissions and 3 children had 3 admissions) were included in the study. The median age was 3 years (range: 0–17 years), 102 (38.3%) children were ≤ 2 years old, and 129 (48.5%) were female (Table 1).

**Figure 1:**
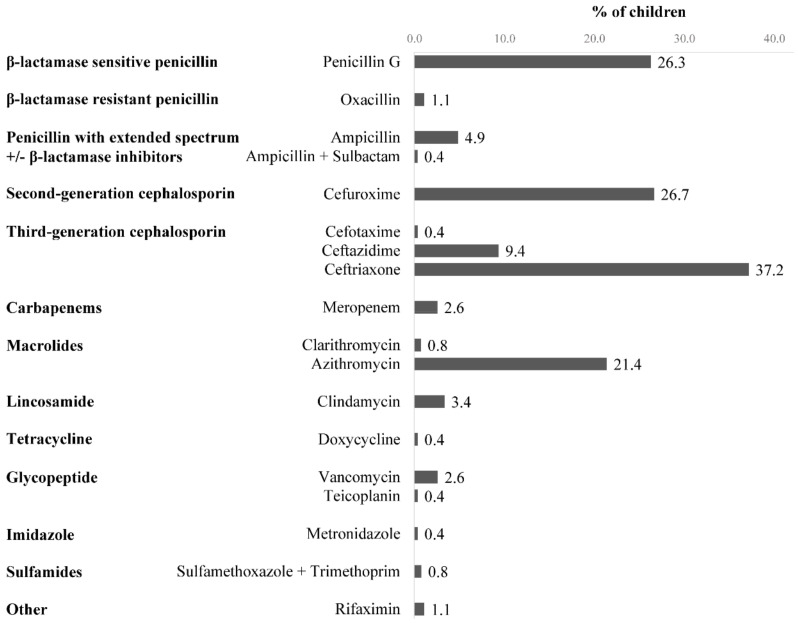
Antibiotics prescribed to children (n = 266) during hospitalization.

Ceftriaxone was the most prescribed antibiotic (99 of 266 children, 37.2%), followed by cefuroxime (71 of 266, 26.7%) and penicillin G (70 of 266, 26.3%) (Figure 1). Ceftriaxone and cefuroxime were usually administered for empirical treatment in respiratory tract infections due to their effectiveness against a wide range of bacteria. During hospitalization, changes in antibiotic treatment were made for 53 (19.9%) children, of whom 12 (4.5%) had their antibiotic changed in less than 24 h after admission. The antibiotic was changed after pathogen identification in 10 children and due to an ADR in one child. The treatment included ≥2 concomitant antibiotics in 46 (17.3%) children. Respiratory tract infections were the main indication for antibiotic use, in 153 (57.5%) children (Table 1).

Overall, 25 (9.4%) children experienced 30 ADRs related to antibiotics. Eighteen ADRs (60.0% of the total ADRs) were recorded in 13 children aged ≤ 2 years (5 experienced 2 ADRs), 11 ADRs (36.7% of the total ADRs) in 11 children aged 3–11 years and 1 ADR in the 12–17-year age group (i.e., elevated transaminases in one 14 year old child). Gastrointestinal (11 of 30, 36.7%) and hematological ADRs (11 of 30, 36.7%) were most frequently reported (Table 2). Four children (1.5% of all children included in the study) experienced skin reactions.

Ceftriaxone was the drug most frequently responsible for ADRs (16 of 30, 53.3%), followed by cefuroxime, ceftazidime and azithromycin (3 of 30, 10.0% each). One child received a higher dose of antibiotic (i.e., ceftriaxone) than the maximum recommended dose. No drug–drug interactions involving antibiotics were found. Twenty-eight (93.3%) ADRs were caused by a single and 2 (6.7%) ADRs by 2 co-administered antibiotics.

Upon evaluating causality, 2 ADRs (6.7%) were considered definite, 12 (40.0%) probable and 16 (53.3%) possible. All gastrointestinal ADRs were classified as probable while all hematological ADRs as possible, except a case of eosinophilia (meropenem) that was classified as probable. Most (28 of 30, 93.3%) of the ADRs were not avoidable. One ADR (diarrhea) following administration of ceftriaxone was classified as definitely avoidable because the antibiotic was prescribed in a confirmed viral infection. An ADR (erythematous maculopapular eruption) was considered possibly avoidable due to the known history of allergy to the administered antibiotic (cefuroxime). Most of the ADRs (19 of 30, 63.3%) were classified in the lowest category of the severity scale and did not require changes in the antibiotic treatment (Table 3). However, 7 (28.0%) children required treatment for their ADRs. Among these, 5 developed diarrhea after administration of ceftriaxone, ceftriaxone + azithromycin and meropenem. Two children developed mycotic stomatitis that needed topical treatment. The antibiotic was withdrawn in 3 (12.0%) children due to ADRs. These were all skin reactions (erythematous maculopapular eruption), and the discontinued antibiotic was ceftazidime, cefuroxime and penicillin G. All patients recovered without sequelae. No death was recorded. 

## 3. Discussion

Broad-spectrum antibiotics are active against gram-positive bacteria and gram-negative bacteria (i.e., glycopeptides [gram-positive only], ampicillin +/− sulbactam, clindamycin, metronidazole, sulfonamides, rifaximin, cefuroxime, third generation cephalosporins), but also against other microorganisms, such as atypical bacteria and mycobacteria (i.e., macrolides and tetracyclines). In this study, third generation cephalosporins were the most frequently prescribed due to their effectiveness in empiric (i.e., central nervous system infections, genitourinary tract infections, bone and joint infections, community-acquired pneumonia, and skin/soft tissue infections) and specific therapy (i.e., gram-negative meningitis and osteomyelitis, Lyme disease, *Pseudomonas pneumonia*, gram-negative sepsis, Streptococcal endocarditis, melioidosis, *Neisseria gonorrhea* and chancroid). Narrow-spectrum antibiotics represented only 27.4% of prescriptions in this study [14,15,16,17,18,19,20,21,22,23]. Penicillin G and oxacillin were prescribed, which are active against gram-positive bacteria [14].

We observed ADRs in 9.4% of children treated with antibiotics. Our result is consistent with data from a previous prospective observational study conducted in pediatric inpatients (12%), but represents a lower frequency of ADRs than that estimated (22.5%) in a systematic review of 83 randomized controlled trials evaluating ADRs to antibiotics in children [24,25]. This difference could be explained by the antibiotic classes used and class-specific ADRs. Cephalosporins were the most frequently prescribed in our study, while penicillins +/− β-lactamase inhibitor were the most frequently administered in the randomized controlled trials included in the review. Furthermore, the review showed a higher risk of diarrhea for the combinations of penicillins and β-lactamase inhibitor compared with other β-lactams (risk ratio 2.4, 95% confidence interval [CI] 1.8–3.2), while this was lower for cephalosporins compared with other β-lactams (risk ratio 0.6, 95% CI 0.4–1.0) [25]. Aminoglycosides, known for their class-specific ADRs (i.e., nephrotoxicity and ototoxicity), were not prescribed at all in our study, but were the second most prescribed antibiotic class (35.6%) in the above mentioned review [25]. Most ADRs in our study (96.7%) occurred in children ≤ 11 years of age, which is consistent with the frequency of ADRs to antibiotics (87.4%) reported in the same age category in a retrospective study [26].

Gastrointestinal and hematological ADRs were the most common in our study. In contrast, previous studies reported dermatological ADRs as being more frequent [26,27]. These differences in ADR types among studies may be due to variation in the antibiotics prescribed, healthcare systems or sample size. Ceftriaxone was the most prescribed antibiotic in our study and was therefore expected to be most frequently associated with ADRs (53.3%). Two ADRs (diarrhea and transaminase increase) were reported when it was co-administered with another antibiotic (azithromycin). The risk of ADRs after antibacterial combination is high, and identifying the precise drug causing the reaction could be difficult for some ADRs (e.g., diarrhea, nephrotoxicity and coagulopathy) [28].

Diarrhea was among the most frequently reported ADRs, especially in children aged ≤ 2 years (6 of 19 ADRs, 33.3%). Baù et al. found a higher risk of diarrhea related to antibiotic treatment in children ≤ 3 years of age compared to older children (relative risk 4.25, 95% CI 2.49–7.27) [29].

Hematological abnormalities were frequently observed in our study. However, on causality assessment, almost all of these ADRs were considered only as possibly related to antibiotics, because the impact of the infection on the hematological changes could not be excluded. Vardakas et al. showed that hematological abnormalities were frequently reported during antibiotic therapy, but most abnormalities resulted as a consequence of the infection and were not antibiotic-induced [30]. In our study, 6.7% of ADRs were classified as definitely and 40.0% of ADRs as probably related to antibiotic use. A study which evaluated the causality of the ADRs related to different drug classes in pediatric inpatients using Naranjo’s algorithm, reported 0.6% of ADRs as definite and 98.1% as probable [31]. Similarly, Khan et al. reported 93.7% of ADRs as probable [26]. The difference in the findings could be explained by the drugs associated with the ADRs and by the different methods used for the causality assessment [32]. The majority of ADRs (93.3%) were classified as not avoidable, which is in line with the results of other studies that assessed the ADRs of different drug classes in hospitalized children, even when different methods were used for the assessment of the avoidability [31,33].

In this study, broad spectrum antibiotics, which are associated with an increased risk of ADRs, were frequently prescribed. A study assessing the impact of an antibiotic stewardship intervention in a Romanian hospital reported a decrease in the consumption of all systemic antibiotics. Efforts are required at national level to implement antimicrobial stewardship programs in hospitals for the prevention of ADRs and antibiotics resistance [34].

This study offers an insight into the characteristics of the ADRs related to pediatric antibiotic use during hospitalization in Romania. Due to the limitations of this study being conducted in a single medical unit over a limited period of time and with a small study sample, the study findings cannot be generalized to national or international level. Furthermore, the small sample size could be associated with a low rate of ADRs being recorded. Another limitation of this study is the follow-up period, as some ADRs might occur after discharge, a period of time that is not captured by our study. We evaluated the causality, avoidability and severity of the suspected ADRs using validated tools in pediatric patients. Regarding avoidability, it is possible that not all factors were mentioned in the medical charts or revealed during the interview, which could have resulted in the misclassification of the ADRs’ avoidability.

## 4. Materials and Methods

### 4.1. Study Design and Participants

An observational prospective pilot study was carried out between November 2017 and April 2018, at the peak of infectious diseases incidence in temperate regions of Europe, including Romania [35], in the pediatric department of the Clinical Hospital for Infectious Diseases in Cluj-Napoca, Romania.

Children < 18 years of age who had been administered antibiotic treatment at admission or during hospitalization were consecutively enrolled. Children for whom the information consent was not obtained were excluded from study.

### 4.2. Data Collection

Demographic data (i.e., age, sex, body weight and allergies), medication history, diagnosis at admission and discharge, duration of hospitalization, and all prescribed drugs during hospitalization (i.e., name of drugs, dose, pharmaceutic form, route of administration, frequency, and duration of treatment) were collected. The diagnosis, treatment and laboratory data were extracted from medical charts. The demographic characteristics, and both the occurrence of ADRs and their evolution were obtained from medical charts and by direct interview with children and their parents, conducted by a pharmacovigilance-trained pharmacist. All included children and their parents were interviewed each working day until discharge or transfer.

### 4.3. Data Analysis

The British National Formulary for Children, Micromedex^®^, and the summary of product characteristics were used to check the doses of antibiotics, drug–drug interactions, and potential ADRs [36,37]. An ADR was defined as any unintended and noxious response to a medicine arising within or outside of marketing authorization, including off-label use, overdose, misuse, medication errors, and abuse [38]. All adverse events were recorded and their causality was evaluated using the Liverpool ADR causality assessment tool; causality was categorized as definite, probably, possible or unlikely [32]. ADRs with causality classified as unlikely were not included in this paper. The Liverpool ADR avoidability assessment tool was used to classify the ADRs into one of the following avoidability categories: ‘not avoidable’, ‘possibly avoidable’ and ‘definitely avoidable’ [39]. The severity of ADRs was characterized using the Modified Hartwig and Siegel scale, and classified as mild (severity levels 1 [required no change in treatment] and 2 [drug dosing or frequency changed]), moderate (severity levels 3 [required treatment or drug administration discontinued] and 4 [resulted in patient transfer to higher level of care]) and severe (severity levels 5 [caused permanent harm to the patient or significant hemodynamic instability] and 6 [directly or indirectly resulted in patient death]) [40].

All data was analyzed descriptively. Categorical data were expressed in numbers and percentages, and continuous variables were described using the median with ranges. The results were stratified by age groups (≤2 years, 3–11 years and 12–17 years).

The study was approved by the Ethics committee of the University of Medicine and Pharmacy Iuliu Hatieganu, Cluj-Napoca, Romania and was conducted according to the principles of Good Clinical Practice and the Declaration of Helsinki. Written informed consent was obtained from the parent or legal guardian of each participant before their inclusion in the study.

## 5. Conclusions

This study assessed the pattern of ADRs following administration of antibiotics in Romanian pediatric inpatients. ADRs more frequently affected younger children. Most ADRs were mild and did not require any intervention. Third generation cephalosporins were responsible for most of the ADRs and gastrointestinal disorders related to antibiotic treatment were frequently reported in children. To enhance safe antibiotic use in hospitalized children, physicians should be aware of the risks associated with broad-spectrum antibiotics. Close monitoring of ADRs associated to antibiotic treatment remains important in the pediatric population.

## Figures and Tables

**Table 1:** Demographic characteristics of the children.

Characteristic	Total (N = 266)	With ADRs (N = 25)	Without ADRs (N = 241)
Age (years; number [%])			
≤2	102 (38.3)	13 (52.0)	89 (36.9)
3–11	145 (54.5)	11 (44.0)	134 (55.6)
12–17	19 (7.2)	1 (4.0)	18 (7.5)
Sex			
Female (number [%])	129 (48.5)	15 (60.0)	114 (47.3)
Male (number [%])	137 (51.5)	10 (40.0)	127 (52.7)
Previous drug allergies (number [%])	24 (9.0)	3 (12.0)	21 (8.7)
Absence of drug allergies (number [%])	242 (91.0)	22 (88.0)	220 (91.3)
Diagnoses at admission (number [%]) ^a^			
Respiratory tract infection	150 (56.3)	11 (44.0)	139 (57.7)
Digestive system infection	63 (23.7)	7 (28.0)	56 (23.2)
Skin infection	39 (14.7)	3 (12.0)	36 (14.9)
Nervous system disease	9 (3.4)	3 (12.0)	6 (2.5)
Others	5 (1.9)	1 (4.0)	4 (1.7)
Diagnoses at discharge (number [%]) ^a^			
Respiratory tract infection	153 (57.5)	12 (48.0)	141 (58.5)
Digestive system infection	54 (20.3)	6 (24.0)	48 (19.9)
Skin infection	46 (17.3)	4 (16.0)	42 (17.4)
Nervous system disease	9 (3.4)	2 (8.0)	7 (2.9)
Others	4 (1.5)	1 (4.0)	3 (1.3)
Number of prescribed antibiotics (number [%])			
1	176 (66.2)	12 (48.0)	164 (68.0)
≥2	90 (33.8)	13 (52.0)	77 (32.0)
Duration of the antibiotic treatment (days; median [range])	5 (1–22)	6 (20–22)	5 (1–21)
Number of co-administered medicines (median [range]) ^b^	4 (0–8)	4 (2–7)	4 (0–8)
Length of hospitalization (days; median [range])	5 (2–22)	6.5 (3–22)	5 (2–36)

^a^ only the diagnosis at first admission was included for children with multiple admissions; ^b^ dietary supplements and topical drugs were excluded; N, total number of children in a given group; ADRs, adverse drug reactions; other: cardio-vascular infections, urogenital infections, musculoskeletal infections, otitis media.

**Table 2:** Listing of the ADRs to antibiotics observed in all children and classified by age groups.

Adverse Reaction [n (%)]	Antibiotics Involved (n)	ADRs in All Children (N = 30)	ADRs in Children ≤ 2 Years (N = 18)	ADRs in Children 3–11 Years (N = 11)	ADRs in Children 12–17 Years (N = 1)
**Hepatic system** Transaminases increase ^a^	azithromycin (1), cefuroxime (1), ceftriaxone (1), ceftriaxone + azithromycin (1)	4 (13.3)	3 (16.7)	0 (0.0)	1 (100)
**Gastrointestinal system**					
Diarrhea	ceftriaxone + azithromycin (1) ceftriaxone (6), meropenem (1)	8 (26.7)	6 (33.3)	2 (18.2)	0 (0.0)
Nausea	clarithromycin (1)	1 (3.3)	0 (0.0)	1 (9.1)	0 (0.0)
Stomatitis	ceftriaxone (2)	2 (6.7)	1 (5.6)	1 (9.1)	0 (0.0)
**Hematological system**					
Anemia	rifaximin (1)	1 (3.3)	0 (0.0)	1 (9.1)	0 (0.0)
Leukopenia	penicillin G (1), ceftriaxone (1)	2 (6.7)	0 (0.0)	2 (18.2)	0 (0.0)
Neutropenia	cefuroxime (1), ceftriaxone (1)	2 (6.7)	1 (5.6)	1 (9.1)	0 (0.0)
Thrombocytosis	ceftriaxone (4), ceftazidime (1)	5 (16.7)	4 (22.2)	1 (9.1)	0 (0.0)
Eosinophilia	meropenem (1)	1 (3.3)	1 (5.6)	0 (0.0)	0 (0.0)
**Skin** Erythematous maculopapular eruption	penicillin G (1), cefuroxime (1), ceftazidime (2)	4 (13.3)	2 (11.0)	2 (18.2)	0 (0.0)

^a^ increased alanine aminotransferase or aspartate aminotransferase or both; N, total number of ADRs; n (%), number (percentages) of ADRs in each category; ADRs, adverse drug reactions.

**Table 3:** Causality, avoidability and severity of the 30 ADRs of antibiotics.

	All ADRs (N = 30)	ADRs in Children ≤ 2 Years (N = 18)	ADRs in Children 3–11 Years (N = 11)	ADRs in Children 12–17 Years (N = 1)
**Causality (n [%])**				
Definite	2 (6.7)	1 (5.6)	1 (9.1)	0 (0.0)
Probable	12 (40.0)	8 (44.4)	4 (36.4)	0 (0.0)
Possible	16 (53.3)	9 (50.0)	6 (54.5)	1 (100)
**Avoidability (n [%])**				
Definitely avoidable	1 (3.3)	1 (5.6)	0 (0.0)	0 (0.0)
Possibly avoidable	1 (3.3)	0 (0.0)	1 (9.1)	0 (0.0)
Not avoidable	28 (93.3)	17 (94.4)	10 (90.9)	1 (100)
**Severity level (n [%])**				
1 (Required no change in treatment)	19 (63.3)	11 (61.1)	7 (63.6)	1 (100)
2 (Drug dosing or frequency changed)	1 (3.3)	1 (5.6)	0 (0.0)	0 (0.0)
3 (Required treatment or drug administration discontinued)	10 (33.4)	6 (33.3)	4 (36.4)	0 (0.0)
4 (Resulted in patient transfer to higher level of care)	0 (0.0)	0 (0.0)	0 (0.0)	0 (0.0)
5 (Caused permanent harm to patient or significant hemodynamic instability)	0 (0.0)	0 (0.0)	0 (0.0)	0 (0.0)
6 (Directly or indirectly resulted in patient death)	0 (0.0)	0 (0.0)	0 (0.0)	0 (0.0)

N, total number of ADRs; n (%), number (percentages) of ADRs in each category; ADRs, adverse drug reactions.

## Data Availability

Data is contained within the article.
